# Nonsteroidal Anti-Inflammatory Drugs (NSAIDs) Use Is Associated With an Increased Risk of Femoral Osteonecrosis in Migraine Patients: A Retrospective, TriNetX Database-Based Study

**DOI:** 10.7759/cureus.95391

**Published:** 2025-10-25

**Authors:** Joseph Bisiani, Francisco Matos Jr., Brian Lynch

**Affiliations:** 1 Department of Orthopaedics, Stony Brook University, Stony Brook, USA

**Keywords:** avascular osteonecrosis, femur, migraine treatment, (nsaid) nonsteroidal anti-inflammatory drugs, prostaglandin

## Abstract

Background and objective

Many patients across the United States suffer from migraines, which can be extremely painful and often require therapeutic management. Nonsteroidal anti-inflammatory drugs (NSAIDs) are among the most commonly prescribed medications for migraine treatment. However, these drugs may carry a potential risk of femoral osteonecrosis, as NSAID use can contribute to microthrombi formation in certain blood vessels and reduced blood flow. Therefore, further research is needed to determine whether the use of these anti-migraine medications is associated with an increased risk of developing femoral osteonecrosis. This study aimed to evaluate whether the use of common NSAIDs for the treatment of migraine is associated with an increased risk of osteonecrosis of the femur.

Methods

A retrospective analysis was conducted using the TriNetX database. The first cohort included patients aged 18 to 50 who had a diagnosis of migraine, no history of femoral neck fractures, and were prescribed any of the following NSAID medications: naproxen, aspirin, diclofenac, or ibuprofen. The second cohort consisted of patients from the TriNetX database who had migraines but were not prescribed any of the specified NSAID medications and had no history of femoral neck fractures. Comparable cohorts were also created and stratified by sex. Propensity score matching was performed to assess the most common risk factors associated with femoral osteonecrosis. The relative risk and odds ratio (OR) for outcomes of femoral osteonecrosis were compared between the two cohorts.

Results

Our findings revealed that across the overall cohort, as well as the male-only and female-only cohorts, there was a statistically significant difference in osteonecrosis outcomes between migraine patients who used NSAIDs and those who did not, indicating clinical significance. In the generalized cohort, anti-migraine medication use was associated with a significantly increased risk of femoral osteonecrosis (risk ratio (RR) = 3.606, 95% confidence interval (CI): 3.112-4.179; OR = 3.609, 95% CI: 3.114-4.182; p < 0.0001). Similarly, stratified analyses showed significant findings in males (RR = 3.897, 95% CI: 2.986-5.086; OR = 3.902, 95% CI: 2.989-5.094) and females (RR = 3.441, 95% CI: 2.872-4.122; OR = 3.443, 95% CI: 2.874-4.124; all p < 0.0001).

Conclusions

These findings are concerning, given the widespread use of NSAIDs for migraine management, as they may contribute to reduced blood flow to the femur and eventual ischemia.

## Introduction

Many individuals across the United States suffer from migraines, which can be intensely painful and often require therapeutic intervention. Nonsteroidal anti-inflammatory drugs (NSAIDs), such as ibuprofen, naproxen, aspirin, and diclofenac potassium, are some of the most common prescription medications used to treat migraines [1]. However, these drugs may pose a potential risk for femoral osteonecrosis, as NSAID use can promote microthrombi formation within certain blood vessels and reduce overall blood flow. Migraines, in particular, are one of the more common neurological disorders that patients develop, affecting approximately 14% of adults in the United States [2]. By competitively inhibiting the binding of arachidonate to both cyclooxygenase (COX) isoenzymes, COX-1 and COX-2, NSAIDs decrease prostaglandin synthesis, producing their characteristic analgesic and anti-inflammatory effects [3].

Recent evidence shows that a prostaglandin known as “prostacyclin” is one of the main prostaglandins synthesized by the blood vessel wall and may play an important role in limiting platelet-mediated thrombosis [4]. If prostacyclin levels are reduced by medications such as naproxen or other NSAIDs, the likelihood of microthrombi formation may increase, particularly in bones like the femur. This is because a femoral head osteonecrosis can involve an alteration of the vascularization of the fine blood vessels irrigating the anterior and superior part of the femoral head [5], thereby making the femur a very susceptible bone to possible microthrombi and ischemia. Therefore, NSAID use for migraine treatment may be linked to an elevated risk of femoral osteonecrosis, as these medications can increase microthrombi formation due to diminished prostacyclin synthesis from vessel walls.

Osteonecrosis, also known as avascular necrosis, is characterized by the necrosis of bone tissue resulting from impaired blood supply, most commonly affecting the head of the femur. The established risk factors include corticosteroid use, trauma, systemic lupus erythematosus (SLE), and chronic alcohol use, among others. Femoral head osteonecrosis has significant clinical implications, frequently resulting in worsening pain, reduced mobility, and ultimately the need for hip replacement surgery. Although some cases are classified as idiopathic, it remains important to explore other potential causes and contributing factors.

In addition, NSAIDs play a crucial role in the overall pain management of osteonecrosis as well. These medications are employed to alleviate pain and reduce inflammation associated with the condition, providing symptomatic relief for individuals affected by avascular necrosis [6]. Therefore, if naproxen indeed exacerbates the underlying mechanisms that contribute to avascular necrosis, as hypothesized, this could fundamentally alter current perspectives on the treatment of osteonecrosis and overall pain management for this condition. This study aimed to examine the potential association between the use of naproxen, aspirin, ibuprofen, or diclofenac and the incidence of femoral head osteonecrosis in migraine patients, compared to those who did not use NSAIDs. The TriNetX database was utilized to evaluate whether these medications are associated with femoral head avascular necrosis.

## Materials and methods

The study cohorts were defined using the International Classification of Diseases (ICD) code for migraines (G43). The first cohort, or exposure group, included patients aged 18-50 years from the TriNetX network after January 1, 2010, who had migraines and were prescribed one of the following anti-migraine medications: aspirin, naproxen, diclofenac, or ibuprofen. The second cohort, serving as the comparison group, comprised patients from the TriNetX network after January 1, 2010, who had migraines but had not been prescribed any of these medications.

**Figure 1:**
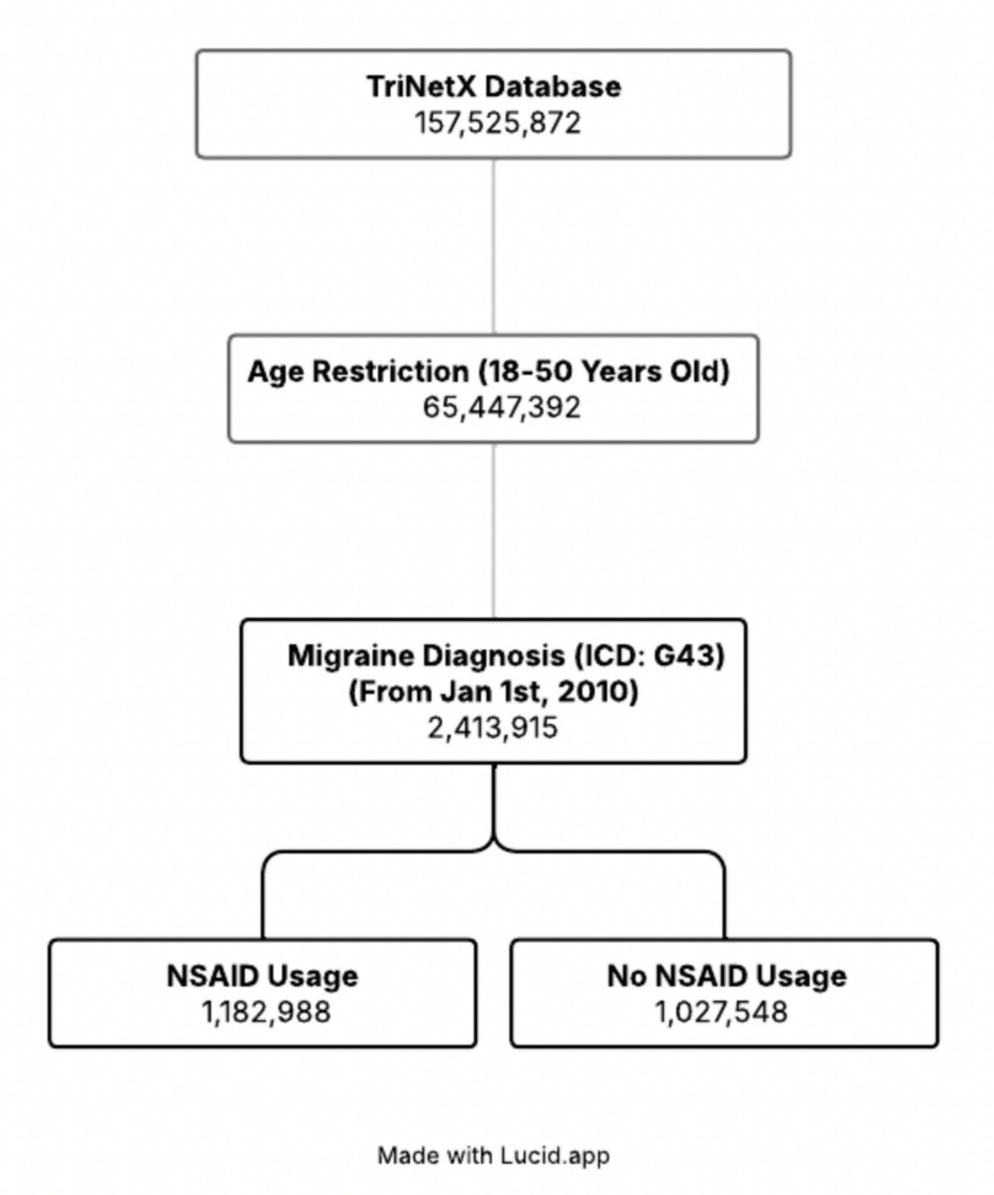
Flow diagram depicting patient cohort selection ICD: International Classification of Diseases; NSAID: nonsteroidal anti-inflammatory drug

Moreover, we excluded patients with any ICD diagnosis of femoral head fractures from both cohorts to remove individuals who may have developed femoral osteonecrosis due to prior trauma. The age range of 18-50 years was selected to capture early adults through middle age, encompassing the peak incidence of migraines, which occurs between 35 and 45 years [7]. The primary outcome evaluated was femoral osteonecrosis. Figure 1 presents a flow diagram illustrating the cohort selection process and the corresponding population sizes at each stage.

Propensity matching was performed to assess the most common cause of osteonecrosis of the femur, as well as any possible confounding factors that may alter the results due to increased risk of avascular necrosis. These include sickle-cell disorders, SLE [8], Gaucher disease, alcohol dependence, nicotine dependence, osteonecrosis due to previous trauma, hyperlipidemia (unspecified), and corticosteroid use. For example, SLE is not only recognized as a contributor to migraines in patients [8] but also serves as a confounding factor for osteonecrosis. Matching was also performed continuously for age and by sex. For sex-specific cohorts, the same approach was applied, except that propensity matching for sex was omitted: one set included only males across the two comparative cohorts, and another included only females. This methodology ultimately produced three analyses: one combining both sexes, one for males only, and one for females only.

Propensity score matching results demonstrated that while most characteristics were well balanced between cohorts, not all achieved statistical equivalence at the conventional threshold of p < 0.05. One limitation of the study design is that some covariates were not significantly different before matching, or, in some cases, became more imbalanced after matching (with lower p-values). Such findings reflect the inherent nature of propensity matching in large databases, where balancing across a broad set of covariates may inadvertently introduce random imbalances in individual characteristics. Table 1 shows the p-values and standardized mean differences for each covariate, both pre-matching and post-matching.

**Table 1:** P-values and standardized mean differences for covariates, before and after matching P-values and standardized mean differences are reported both before and after matching, and are provided from the TriNetX platform. TriNetX does not disclose the specific statistical tests or underlying test statistics that are used to generate the p-values. Therefore, these cannot be reported

Characteristics	P-value before matching	Std. Diff. before matching	P-value after matching	Std. Diff. after matching
Age at index	< 0.0001	0.1381	< 0.0001	0.0280
Male	< 0.0001	0.1296	0.8047	0.0004
Female	< 0.0001	0.1325	0.9816	< 0.0001
Hyperlipidemia, unspecified (E78.5)	< 0.0001	0.2046	0.8966	0.0002
Personal history of nicotine dependence (Z87.891)	< 0.0001	0.2804	0.9728	< 0.0001
Alcohol dependence (F10.2)	< 0.0001	0.1024	0.9342	0.0001
Corticosteroids (ATC: R01AD)	< 0.0001	0.6364	0.9631	< 0.0001
Osteonecrosis due to previous trauma, left femur (M87.252)	0.0020	0.0043	0.0016	0.0050
Osteonecrosis due to previous trauma, right femur (M87.251)	0.7526	0.0004	1.0000	< 0.0001
Systemic lupus erythematosus (SLE) (M32)	< 0.0001	0.0858	0.5914	0.0009
Sickle-cell disorders (D57)	< 0.0001	0.0990	0.7979	0.0004
Gaucher disease (E75.22)	< 0.0001	0.0089	0.0542	0.0031

For outcome analysis, any ICD diagnosis of idiopathic aseptic necrosis of the left femoral head, right femoral head, or an unspecified femur was considered a study outcome. Outcomes were defined as those occurring after a diagnosis of migraine and exposure to NSAID therapy. By anchoring outcomes to the index event of NSAID use in patients with migraine, we ensured that cases of femoral osteonecrosis represented new-onset disease, rather than recurrences.

After matching, the total number of patients in the generalized cohorts was 793,197 individuals each. Meanwhile, the total number of patients in the male cohorts was 153,458 individuals each, and the total number of patients in the female cohorts was 618,680 individuals each. TriNetX outcomes were analyzed with data current as of August 27, 2025.

## Results

Our findings demonstrated that in the generalized, male-only cohorts and female cohorts, there was a statistically significant difference between osteonecrosis outcomes between the two migraine patient cohorts, with clinically significant findings. The risk ratios, odds ratios, and corresponding p-values for the femoral osteonecrosis outcomes are displayed below in Table 2.

**Table 2:** Risk ratios, odds ratios, and corresponding p-values for femoral osteonecrosis by sex Risk ratios (RR) and odds ratios (OR) are presented with 95% confidence intervals (CIs) and p-values all generated by the TriNetX platform. All calculations are performed internally by TriNetX. Test statistics are not provided by the platform

Cohort	Risk ratio (95% CI)	Odds ratio (95% CI)	P-value
Generalized	3.606 (3.112, 4.179)	3.609 (3.114, 4.182)	< 0.0001
Male-only	3.897 (2.986, 5.086)	3.902 (2.989, 5.094)	< 0.0001
Female-only	3.441 (2.872, 4.122)	3.443 (2.874, 4.124)	< 0.0001

For the generalized cohorts, the medication group had an RR of 3.606 (95% CI: 3.112, 4.179) and an OR of 3.609 (95% CI: 3.114, 4.182) for developing osteonecrosis of the femur, compared to the group that did not take the NSAID anti-migraine medications. Meanwhile, in the male-only analysis, the results demonstrated that males in the medications cohort had an RR of 3.897 (95% CI: 2.986, 5.086) and an OR of 3.902 (95% CI: 2.989, 5.094) for developing osteonecrosis of the femur, compared to the males who did not take the NSAID medications. For the female-only analysis, the results demonstrated that females in the medications cohort had an RR of 3.441 (95% CI: 2.872, 4.122) and an OR of 3.443 (95% CI: 2.874, 4.124) for developing osteonecrosis of the femur, compared to the females who did not take the NSAID medications. All of these findings were statistically significant (p < 0.0001).

Across all three comparative analyses, migraine patients treated with commonly used NSAIDs (naproxen, aspirin, ibuprofen, or diclofenac) exhibited a statistically significant increase in the risk of developing femoral osteonecrosis compared with migraine patients who did not use these medications. Clinically, this translates to at least a threefold increase in risk associated with NSAID exposure. 

Regarding overall outcomes, femoral osteonecrosis occurred in 815 patients in the generalized NSAID cohort compared with 226 patients in the non-NSAID cohort. In males, the NSAID cohort had 265 cases of femoral osteonecrosis, compared with 68 cases in the non-NSAID cohort. Among females, the NSAID cohort had 523 cases, while the non-NSAID cohort had 152 cases. Overall, the female cohort experienced approximately twice as many femoral osteonecrosis cases as the male cohort. The total number of outcomes for each cohort and sex is presented in Table 3.

**Table 3:** Femoral osteonecrosis outcomes by cohort and sex NSAID: nonsteroidal anti-inflammatory drug

Cohort type	Generalized outcomes of femoral osteonecrosis	Males-only outcomes of femoral osteonecrosis	Females-only outcomes of femoral osteonecrosis
NSAID usage	815	265	523
No NSAID usage	226	68	152

## Discussion

The results indicate a statistically significant association between the use of naproxen as an anti-migraine NSAID and the development of femoral osteonecrosis. In our cohort of patients aged between 18-50 years old suffering from migraines, those who used the anti-migraine medication NSAID had a modestly increased risk of developing osteonecrosis of the femoral head as opposed to those who did not use the medications. This stayed true across sex-specific analyses, with increased risk ratios for male and female patients of 3.897 and 3.441, respectively.

The results of the study raise concern, given the widespread use of naproxen, aspirin, diclofenac, or ibuprofen to treat migraines. While it has not been fully studied, the effects of naproxen on microthrombi development due to diminished prostacyclin synthesis could be the cause of impaired blood flow to the femur, ultimately resulting in osteonecrosis. With increased microthrombi being produced in an extremely susceptible area, such as the femur, the higher risk ratio seen for femoral osteonecrosis in migraine patients who take naproxen makes sense on a physiological level. While this hypothesis is consistent with the pathophysiology of osteonecrosis, again, a direct causal relationship has yet to be established.

The association between NSAID anti-migraine medications and osteonecrosis still holds despite propensity matching for other risk factors of osteonecrosis, such as alcohol and nicotine use, sickle-cell disorders, SLE, and corticosteroid use, among others. This strengthens the argument that NSAID anti-migraine medications may play a significant role in the progression of osteonecrosis, although there are still some confounders that have yet to be studied. These confounders include dose, duration, comorbid metabolic factors, or genetic predispositions. Additionally, a limitation of this study is that the propensity matching did not account for typical use of naproxen, ibuprofen, diclofenac, or aspirin, as these NSAIDs are commonly used to treat a variety of other conditions. If any of these conditions are potentially associated with femoral osteonecrosis, this could introduce inherent bias into the study design.

While the absolute risk increase varied between male and female cohorts, the widespread use of naproxen highlights its broader clinical significance. Given that millions of patients take NSAIDs such as naproxen, even a threefold increase in relative risk of femoral osteonecrosis carries important clinical ramifications. One in seven American adults suffers from migraines [9], and naproxen was the 103rd most prescribed drug in the United States in 2023, with over 6.8 million prescriptions being filed [10]. One study even demonstrated that roughly a third of patients suffering from migraines have relied on acute prescription medication [11] (which could plausibly include naproxen). It can therefore be estimated that a significant portion of patients may have already been affected by negative outcomes of femoral osteonecrosis after taking anti-migraine naproxen medications. Given the widespread use of other NSAIDs like aspirin and ibuprofen, the observed numbers may be even greater than this.

These statistically significant findings highlight the relative risk of femoral osteonecrosis associated with NSAID use in migraine patients and may have important implications for clinical management. NSAIDs are traditionally a cornerstone of symptomatic treatment in osteonecrosis, providing pain relief and reducing inflammation, even chronically [12]. If agents like naproxen or other NSAIDs do contribute to the development of osteonecrosis, this would challenge current treatment paradigms and highlight the need to explore alternative approaches for pain control and disease management in affected patients who have avascular necrosis.

For instance, regarding the general effects NSAIDS have on bone health and healing, previous studies have found that NSAID use was not associated with a significant change in the risk of delayed healing [13]. In addition, general supplemental pain relief for knee and hip osteoarthritis often involves NSAID therapy [14]. NSAIDs have been employed for a wide range of orthopedic conditions, with minimal concerns regarding bone health or delayed healing, emphasizing their positive pain-relieving factors. Yet, the findings from this TriNetX analysis bring to light some possible subliminal increased risks from overuse of these medications in patients who suffer from migraine. With possible microthrombi development in the femoral vasculature and diminished blood flow to the femur, chronic NSAID usage could be a contributing factor to osteonecrosis.

Interestingly, recent research has begun to investigate potential long-term side effects of NSAIDs that were previously underappreciated. A 2022 study examined prolonged NSAID use and its subtle impacts on the cardiovascular system, concluding that COX inhibitors should be administered at the lowest effective dose for pain relief, particularly in individuals with elevated cardiovascular risk [15]. In addition, new research from placebo-controlled trials and meta-analyses has amplified concerns regarding the effects of NSAIDs on the hepatic and renal systems [16]. Therefore, the association between chronic NSAID use and femoral osteonecrosis represents yet another potential complication to emerge over the past few decades. It is plausible that microthrombi formation from chronic NSAID use contributes to an increased risk of femoral osteonecrosis, particularly given the long-term reliance on these medications among many migraine patients.

This study has several limitations. First, it is based on a single database, which poses inherent challenges in tracking patients longitudinally. Using the TriNetX platform requires that patients receive both neurological (migraine) and orthopedic (osteonecrosis) care within participating institutions, which may not capture care delivered elsewhere. Second, the analysis relies on ICD coding, which may vary in accuracy and consistency across the hundreds of health systems contributing to TriNetX. Third, important confounders such as medication adherence, dosage, and duration of use could not be assessed. This is particularly relevant for NSAIDs such as naproxen and ibuprofen, which are widely available over the counter and may be used for multiple conditions beyond migraine, introducing additional confounding. Finally, the widespread use of these medications limits the generalizability of our findings. Future prospective studies should incorporate prescription data, dose-response analyses, and verification from multiple sources regarding NSAID usage among migraine patients at a national level to validate and expand upon these results.

## Conclusions

In this large, propensity-matched cohort study, patients using common NSAIDs, such as ibuprofen, naproxen, aspirin, or diclofenac, for migraine treatment exhibited an increased risk of femoral osteonecrosis compared to patients not taking these medications. This association was observed consistently in both male and female subpopulations. The widespread use of these medications could potentially impact clinical outcomes of avascular femoral necrosis for patients with migraine. Clinicians should be aware of the increased risk of osteonecrosis when prescribing these medications, especially in patients with comorbid conditions. Further research is needed to explore causality, underlying pathophysiology, and strategies to mitigate this risk.
